# Weakly Coupled Piezoelectric MEMS Resonators for Aerosol Sensing

**DOI:** 10.3390/s20113162

**Published:** 2020-06-02

**Authors:** Malar Chellasivalingam, Hassan Imran, Milind Pandit, Adam M. Boies, Ashwin A. Seshia

**Affiliations:** Department of Engineering, University of Cambridge, Cambridge CB2 1PZ, UK; mc2076@cam.ac.uk (M.C.); hi243@cam.ac.uk (H.I.); milind1993@gmail.com (M.P.); amb233@cam.ac.uk (A.M.B.)

**Keywords:** particulate matter, soot particles, coupled MEMS resonators, amplitude ratio stability, frequency stability, amplitude ratio sensitivity, piezoelectric MEMS, Q factor

## Abstract

This paper successfully demonstrates the potential of weakly coupled piezoelectric MEMS (Micro-Electro-Mechanical Systems) gravimetric sensors for the detection of ultra-fine particulates. As a proof-of-principle, the detection of diesel soot particles of 100 nanometres or less is demonstrated. A practical monitoring context also exists for diesel soot particles originating from combustion engines, as they are of serious health concern. The MEMS sensors employed in this work operate on the principle of vibration mode-localisation employing an amplitude ratio shift output metric for readout. Notably, gains are observed while comparing parametric sensitivities and the input referred stability for amplitude ratio and resonant frequency variations, demonstrating that the amplitude ratio output metric is particularly suitable for long-term measurements. The soot particle mass directly estimated using coupled MEMS resonators can be correlated to the mass, indirectly estimated using the condensation particle counter used as the reference instrument.

## 1. Introduction

Piezoelectric MEMS (Micro-Electro-Mechanical Systems) resonators have shown great potential for the development of high resolution and low power physical, chemical, and biological sensors [1,2,3]. Today, AlN (Aluminium Nitride) is the commonly employed material in RF (Radio Frequency) devices based on piezoelectric thin films, e.g., filters, duplexers, and other front-end modules [4,5]. Piezoelectric actuation and sensing of bulk acoustic modes of vibration in AlN-on-Si (Aluminium Nitride-on-Silicon) plate structures are made possible due to the high electromechanical coupling and high-quality factors achievable in these devices. Advances in manufacturing know-how and the potential compatibility with CMOS (Complementary Metal Oxide Semiconductor) technology provide additional benefits for the realisation of resonant sensors based on this platform [6]. Since resonant MEMS based on piezoelectric transduction offers a unique combination of attributes for resonant gravimetric sensing, recent efforts have been focused on implementing chip-scale particulate sensors [7,8,9,10] in this technology. Moving towards that direction, this work addresses the sensing of particulates that are less than 100 nanometres in diameter, and that is primarily generated by combustion processes in diesel and spark-ignition engines [11,12] with a known detrimental impact on the environment.

There is particular concern about the toxic effects of ultra-fine particulate matter (particles less than 100 nm diameter) and, so, much recent attention has been provided to studying the effect of ultra-fine particulates on human health [13,14,15]. The European Environment Agency’s Outlook for 2050 estimates that the worldwide premature deaths from exposure to the particulate matter would be 2.5 million times higher than that of the deaths expected to result from ozone pollution [16]. The WHO suggests that by reducing the particulate matter levels, the public risk to health conditions including stroke, lung cancer, heart diseases, and respiratory diseases, such as asthma would be significantly reduced [17]. As compelling evidence, measurements made near roadways suggest that particulate matter from motor vehicle emissions pose a significant health risk [18]. It is important to realize that the particulate emissions, especially from diesel engines are typically 10–100 times higher than those from spark-ignition engines [19]. It has been noted that diesel exhaust particles consist mainly of highly agglomerated solid carbonaceous material and ash, and volatile organic and sulphur compounds. Because of the concern towards ultra-fine particulate emissions, diesel engines, thus, have been subjected to increasingly stringent tailpipe emission standards and current emission standards are mass-based [20].

In recent years, QCM (Quartz Crystal Microbalance), SAW (Surface Acoustic Wave), FBAR (Film Bulk Acoustic Resonator), and freestanding MEMS devices have been configured as resonant gravimetric sensors for particulate matter (PM) detection. The transduction principle adopted in these studies relies on monitoring the resonant frequency shift as a function of adsorbed mass [21,22,23]. However, the QCM faces difficulties with integration into a single-chip smart microsystem where all components can be co-integrated onto the same substrate. Whereas, SAW, FBAR, and freestanding MEMS devices could exhibit significant frequency drift due to temperature variations and other environmental effects, limiting long term measurements for monitoring PM [24,25]. Although SAW and FBAR can be realized as films deposited onto a substrate and are easier to integrate into on-chip microsystems, they still have limitations due to their mass sensitivities [26]. On the other hand, in capacitive transduction-based PM sensors, the targeted larger sensitivity and resolution would pose a serious challenge as the elimination of parasitic components becomes far from trivial, when the sensor is to be integrated with an ASIC (Application Specific Integrated Circuit) [27,28].

Alternative technologies for particulate matter detection based on light scattering have been commonly employed and the operation of many commercially available low-cost sensors relies on this approach. However, a significant disadvantage of light scattering-based detectors is that the accuracy of measurements is affected by the size and composition of the particles. Furthermore, the detection of the commercially available optical scattering sensors is limited to particles larger than 300 nm [29]. Notwithstanding this, recent works in [30], [31] have attempted to address limitations associated with existing approaches by utilising weakly coupled piezoelectric MEMS resonators for sensing ultra-fine particles. A significant improvement in particulate mass sensitivity by three orders of magnitude compared to resonant frequency shift, and a degree of passive immunity to temperature effects have been demonstrated by using an amplitude ratio output metric, in weakly coupled MEMS resonators. Though there are potential disadvantages associated with the amplitude readout, the enhanced sensitivity based on amplitude ratio readout would enable these sensors to achieve improved input-referred stability. Particularly, for long term PM measurements, the amplitude noise can be reduced by assuming white noise characteristics [32].

In this paper, we have successfully demonstrated the potential of weakly coupled piezoelectric AlN-on-Si square plate bulk acoustic MEMS resonators to detect and quantify diesel soot particles of approximately 100 nanometres in diameter. We have validated the claim of amplitude ratio stability over long integration times and compared it with the frequency stability of the same device. We have also shown that the mass estimation of diesel soot particles using the weakly coupled piezoelectric MEMS resonators can be correlated to that of the mass estimated using a condensation particle counter (CPC), which is used as the reference instrument in this study.

## 2. Materials and Methods

**Figure 1:**
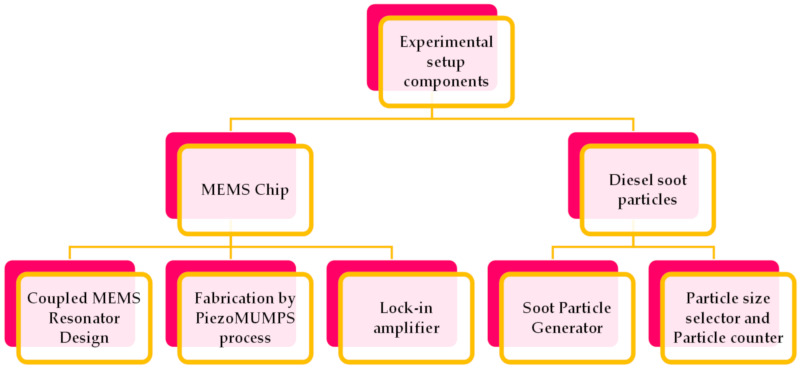
Different components of the experimental study.

The different components comprising this experimental study as illustrated in Figure 1 are:

(a) Coupled MEMS resonator design.
(b) Fabrication of designed MEMS resonators and transduction.
(c) Construction of soot particle generator.
(d) Differential mobility analyser (DMA) and condensation particle counter (CPC).
(e) MEMS Impactor Stage and Measurement electronics.

Section (f) describes the experimental procedure for open-loop frequency response-based soot particle impaction and closed-loop resonator stability experiments.

### 2.1. Coupled MEMS Resonator Design

To demonstrate the vibration mode localisation principle and its enhanced mass sensitivity to aerosols through the weakly coupled resonator approach, two square plate bulk acoustic MEMS resonators operating in their in-plane Lamé mode are designed. As the total volume is conserved in the Lamé mode, and as works in [33,34] have reported higher quality factor for Lamé mode in square plate resonators, coupled Lamé mode is chosen as the desired mode of operation. The square plate resonators are fixed at their four corners by T-shaped anchors, which in turn would minimize anchor losses, as the corners signify the nodal points of Lamé mode. As described in [30], a quarter-wavelength mechanical beam is used as a coupler connecting the two resonators at their nodal points to maximize the effects of mode localisation by providing weak coupling. The dimensions of the weakly coupled piezoelectric MEMS resonators used in this experiment are described in Table 1.

Piezoelectric AlN is stacked on top of the silicon plate such that it encompasses each of the four antinodal points of the desired Lamé mode to enable its actuation and transduction through the piezoelectric effect. Note that the piezoelectric effect allows the transduction of the motional currents arising as the output due to amplitude displacements from individual resonators in the array. The four triangular aluminium electrodes patterned on top of piezoelectric AlN aids the external circuit connections to actuate and sense the desired coupled Lamé mode (vibrating out-of-phase and in-phase) in the resonator array. It should also be noted that the parasitic feedthrough capacitance cancellation could be effectively implemented for the coupled Lamé mode through differential drive configuration [35]. 

### 2.2. Fabrication of Designed MEMS Resonators and Transduction

The designed MEMS resonators are fabricated using silicon-on-insulator (SOI) wafers via a five-mask modified industrial Multi-User MEMS process (MUMPs) by MEMSCAP Inc., as given in [36]. The fabrication process begins by depositing, annealing, and wet etching a phosphosilicate glass layer on top of a double-side polished silicon-on-insulator wafer. This is then followed by thermal oxide growth, piezoelectric AlN film deposition, and Al metal deposition for lithographically patterning the electrode structures. Following this, the backside of the substrate is etched using RIE (Reactive Ion Etching) of the backside oxide, subsequent DRIE (Deep Reactive Ion Etching) of the silicon handle layer followed by wet release of the buried oxide and dry etch of a protective front side material to release the mechanical structures.

To actuate and sense the coupled Lamé modes, two-port transduction is used in which two sets of electrodes could be used for actuation and two sets of electrodes could be used for sensing. An AD8131 amplifier is used in the unity gain configuration to generate the in-phase and out-of-phase signal required for actuating the coupled Lamé modes using a differential drive configuration. Using the differential drive signals (+V_ac_ and −V_ac_) generated, two sets of electrodes in one of the coupled resonators are chosen for actuating the desired coupled Lamé mode. Since the electrodes are independently accessible, the motional current output from each of the resonators is then sensed through any of the electrodes that are not used for actuation.

**Figure 2:**
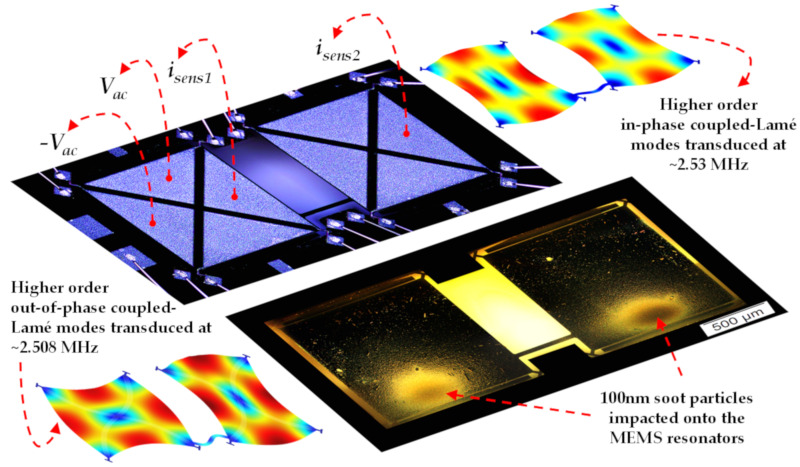
Illustration of the actuation and transduction mechanism used in the coupled MEMS resonators for the out-of-phase and in-phase coupled Lamé modes with the 100 nanometre diameter soot particles impacted onto the resonators.

Thus, the coupled MEMS resonator system is operated in a single resonator drive and dual resonator sense (i_sens1_ and i_sens2_) configuration as shown in Figure 2. The resonator body is grounded at all the four corners of the two square plate resonators through the T-shaped anchors to minimize feedthrough and eliminate the effects of unknown node potential across the piezoelectric films. It should be noted that Figure 2 also depicts the dark-field microscopic image of both the resonators in the array impacted with soot particles. However, in this study, experimental results from soot particle impaction on only one of the coupled MEMS resonators are taken into consideration.

Furthermore, the vibrational response of resonator 1 at the in-phase mode frequency can be orders of magnitude lower than that at the out-of-phase mode frequency, if the coupled MEMS resonator system is driven with only one actuation force (single resonator drive) as reported in [37]. This would also mean that the dominant loss mechanisms in the coupled MEMS resonator array are due to (1) anchor losses that are dependent on cancellation of force at the anchor points [38] when the coupled MEMS resonators vibrate at their out-of-phase and in-phase motion, (2) air/fluidic losses as the resonators are not operated in a vacuum environment, and (3) surface losses due to mass loading.

### 2.3. Construction of Soot Particle Generator

**Figure 3:**
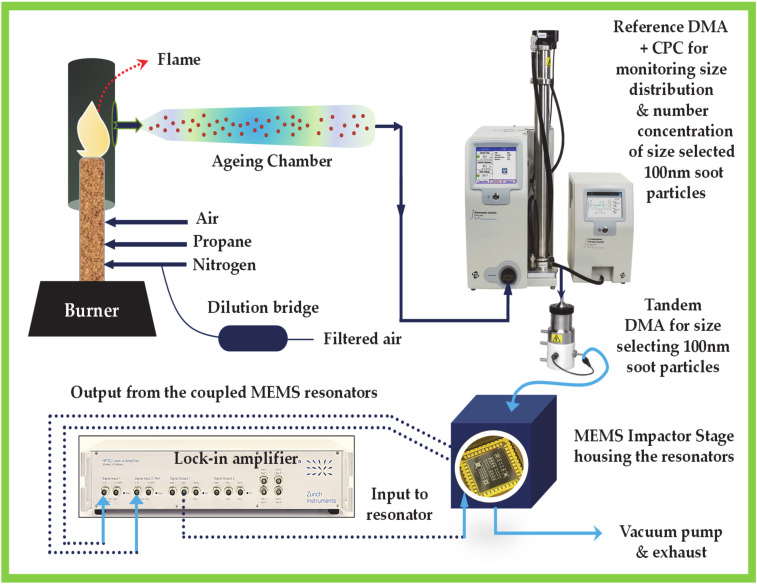
Experimental setup including soot particle generator, MEMS Impactor Stage housing the resonators and associated electronics; DMA stands for Differential Mobility Analyser and CPC stands for Condensation Particle Counter.

The soot particle generator comprises a burner consisting of two co-annular tubes for fuel and co-flow air, respectively [39]. In this experiment, propane (65–105 std.cm^3^/min) is selected as the gaseous fuel source. Stable, carbonaceous soot particles are produced by burning propane (65–105 std.cm^3^/min) with air (1.2 std.L/min) in a co-flow inverse diffusion flame using N_2_ (3 std.L/min) as a sheath flow. The generated inverse diffusion flame is enclosed by a quartz tube and it is stabilised by attaching an annular shaped bluff body at the outlet of the fuel tube. A portion of the co-flow air near the flame is used for the combustion of the fuel while the rest of the co-flow air diluted the combustion products downstream of the flame, followed by an ageing chamber, as shown in Figure 3. A dilution bridge is used to generate different soot particle concentrations, which consists of a HEPA (High Efficiency Particulate Air) filter in parallel. The concentration of the generated soot particles is varied by changing the flow rate of the dilution air. The particle size is varied by changing the flow rate of propane into the flame. Higher flow rates of propane would yield larger particles [40].

### 2.4. Differential Mobility Analyser and Condensation Particle Counter

**Figure 4:**
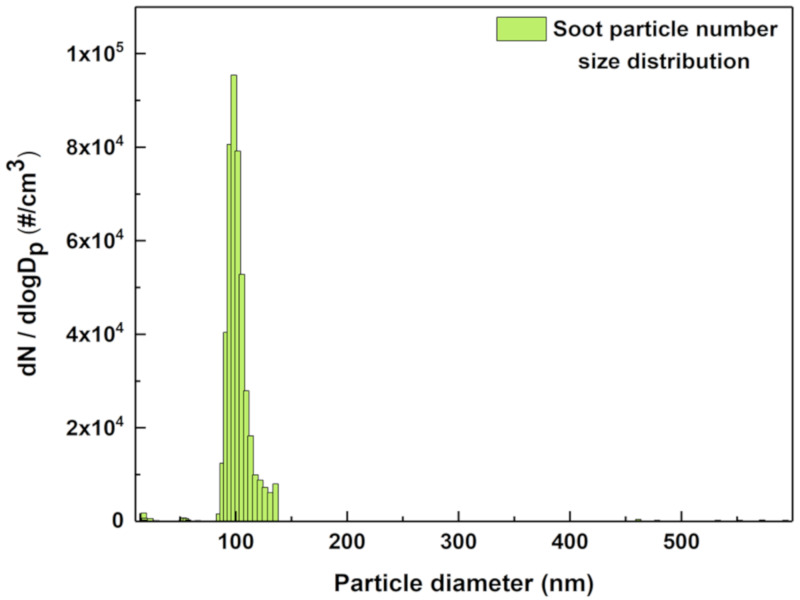
Size distribution of the 100 nm size selected soot particles monitored using the reference Differential Mobility Analyser and Condensation Particle Counter.

For size selecting 100 nm soot particles, a differential mobility analyser (DMA) is connected at the outlet of the ageing chamber, which is then connected to the condensation particle counter (CPC). The size selected soot particles from the DMA are counted by the CPC and are measured in terms of particle number concentration for reference as shown in Figure 4. A tandem DMA arrangement is used in the experiment such that the output of one DMA is connected to the CPC for measuring the size distribution and particle number concentration of the size selected soot particles. Similarly, the output of the other DMA is connected to the MEMS Impactor Stage so that only size selected 100 nm soot particles are collected on the surface of the MEMS resonators housed within the Impactor Stage.

### 2.5. MEMS Impactor Stage and Measurement Electronics

The output or size selected 100 nm soot particles from the DMA is connected to the MEMS Impactor Stage (MIS) housing the weakly coupled resonators. A traditional impactor stage consists of a nozzle and an impaction plate in which the main geometric parameters are the nozzle jet diameter and the separation between the nozzle and the impaction plate. The MIS is a single-stage impactor where the MEMS resonators serve as the impaction plate allowing for the collection of particles, passed through the nozzle jet via inertial impaction. The outlet of the MEMS Impactor Stage is connected to a vacuum pump through a needle valve, which is then routed to the exhaust. The vacuum pump aids in regulating the inlet flow rate through the nozzle that draws the particles onto the MEMS resonators. As a result, the generated soot particles are deposited onto the surface of the MEMS device.

A two-channel lock-in amplifier (HF2LI, Zurich Instruments) is used for monitoring the frequency response of the coupled MEMS resonators (resonator 1 and resonator 2) during soot particle impaction in an open-loop configuration. The frequency response of the coupled MEMS resonators provides information about the shift in amplitude ratio and shift in the resonant frequency of the resonators, which is then correlated directly to the mass of the soot particles accumulated on the resonator surface. The MEMS sensor can provide a direct mass measurement, independent of the particle size as opposed to optical particle counters [41]. Particle density and size variations affect the collection efficiency [42] for the MEMS resonators rather than the actual mass measurement, in distinction to instruments based on light scattering measurements. To investigate the long-term stability of the coupled MEMS resonator system, the amplitude ratio and resonant frequency data from the coupled resonators are obtained for 12 h in a closed-loop configuration.

### 2.6. Soot Particle Impaction and Resonator Stability—Experiment

To demonstrate the enhanced sensitivity of coupled MEMS resonators based on vibration mode localisation to sensing soot particles, the experiment began by collecting soot particles onto one of the two coupled resonators. The typical arrangement within the MIS is to have the MEMS resonators chip attached upside down in a chip holder socket such that particles are collected on the backside-silicon surface of the resonators. Having the chip upside down protects the electrical connections (wire-bonds) and limits particle collection on the transducer electrodes in the front surface of the piezoelectric resonators. The size-selected 100 nm particles pass through the size-selective inlet nozzle with a 50% cut-off efficiency at 100 nm aerodynamic diameter. The flow rate at the inlet of the nozzle is regulated at 0.695 L/min by the vacuum pump at the exhaust.

Inertial impaction of soot particles onto one of the coupled MEMS resonators also involves the alignment of the nozzle onto the resonator surface. The alignment of the nozzle onto the desired location in the resonators is performed by simple crosshairs overlaid using a reconfigurable attachment of USB (Universal Serial Bus) camera and microscope in the MIS by Peek Through (Windows XP application provided by Luke Payne Software, Australia). Initially, a blank silicon substrate is positioned for soot particle deposition. After aligning the nozzle using crosshair onto the blank silicon substrate, soot particles are collected for a few minutes. Images of the blank silicon substrate are taken before and after particle collection. Note that the MIS also has a provision for recording the temperature and humidity data inside the MEMS Impactor volume by external temperature and humidity sensors connected to a data logger.

**Figure 5:**
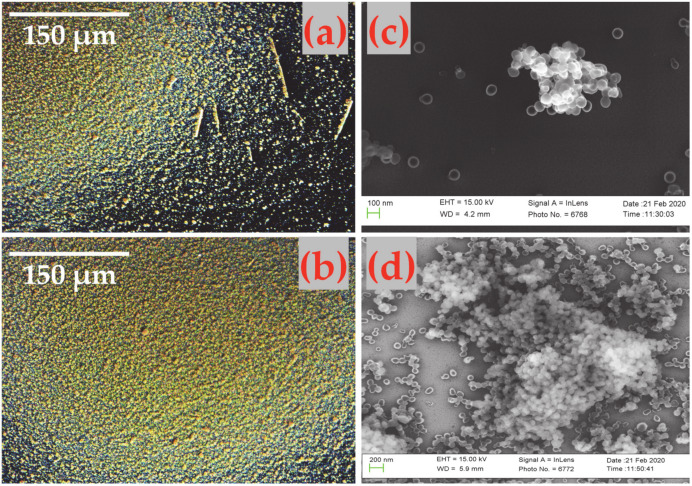
(**a**) and (**b**) Darkfield optical microscopic images, (**c**) and (**d**) SEM images of the soot particles adsorbed on the resonator surface.

Based on the position information obtained from particle deposition on the blank silicon substrate, the nozzle is then aligned to the MEMS resonator surface for particle impaction. It should be noted that for smaller sized particles, the spatial distribution of particles on the resonator surface is affected by the distance between the nozzle and resonator surface and, nozzle shape. To begin with, soot particles are then impacted onto one of the coupled MEMS resonators for 2 min. The frequency response of the coupled MEMS resonators is monitored in an open-loop configuration using the lock-in amplifier before and after soot particle impaction. This experiment is then continued for a total time duration of 40 min at different time intervals and the frequency response of the coupled MEMS resonators are monitored in open-loop after each interval of soot particle impaction. The impaction experiment is performed such that the soot particles are accumulated only onto the antinode positions of the desired coupled mode of transduction. Figure 5 shows the microscopic and SEM images of the soot particles impacted onto one of the coupled MEMS resonators after 40 min of soot particle impaction. The frequency response measurements are recorded for both the out-of-phase and in-phase coupled Lamé modes of vibration using the two-channel lock-in amplifier (HF2LI) after each time interval of soot particle deposition.

The long-term stability measurements for the weakly coupled MEMS resonators are obtained by monitoring the amplitude ratio and resonant frequency data by establishing a phase-locked loop (PLL) for the out-of-phase mode centred around 2.508 MHz. During this closed-loop experiment for recording long-term stability of amplitude ratio and frequency, soot particles are not deposited onto the resonators. In a symmetrical system, the out-of-phase mode stability can be assumed to be similar to the in-phase mode stability [32]. The Allan deviation of the amplitude ratio and resonant frequency of the coupled MEMS resonators at the out-of-phase Lamé mode is then calculated to compare the stability of the two-output metrics.

## 3. Theory and Modelling of the Coupled MEMS Resonators

**Figure 6:**
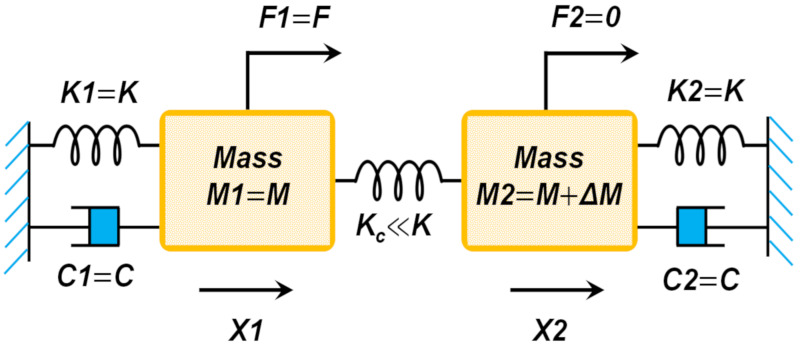
Mass spring damper representation of the coupled MEMS resonator system when a mass perturbation (*ΔM*) is introduced in one of the coupled resonators.

Weakly coupled piezoelectric MEMS resonators used in this work are designed around the principle of vibration mode localisation as described in [43,44]. In a system of weakly coupled MEMS resonators, symmetry-breaking perturbations result in the confinement of vibration energy in certain spatial locations due to the principle of vibration mode localisation. A practical realisation may involve a system of two coupled resonators with vibration energy confinement being monitored, by independently measuring the responses of each of the individual resonators separately and evaluating the amplitude ratio. For a symmetric system, the eigenstates would be correspondingly symmetric, and this is reflected in the measurement of the amplitude ratio output metric. If even a small perturbation is then applied to the system, by either varying the mass or varying the stiffness, the symmetry of the system breaks, and vibration modes in the system will localize. This variation in mass or stiffness could be created by adding or removing mass on one or both the resonators in the array. The resulting vibration energy confinement is recorded through the ratio of the amplitudes of the motional current variations from individual resonators in the array. The two degrees of freedom system denoting the coupled MEMS resonator can be represented as shown in Figure 6.

The vibration mode localisation phenomenon can be modelled using this simple discrete model of two degrees of freedom resonator system and the equations of motion for this system is given by:

(1) $$M_{1}\ddot{X_{1}}+C_{1}\dot{X_{1}}+K_{1}X_{1}+K_{c}\left(X_{1}-X_{2}\right)=F_{1}$$

(2) $$M_{2}\ddot{X_{2}}+C_{2}\dot{X_{2}}+K_{2}X_{2}+K_{c}\left(X_{2}-X_{1}\right)=F_{2}$$

On solving these equations of motion by assuming initial conditions as described in [30], the modal frequencies and amplitude ratio at each mode of vibration (out-of-phase and in-phase) for the coupled MEMS resonators can be obtained as,

(3) $$\omega_{1}^{2}=\frac{\left(K+K_{c}\right)\left(∆M+2M\right)+\sqrt{∆M^{2}{\left(K+K_{c}\right)}^{2}+4MK_{c}^{2}\left(∆M+M\right)}}{2M\left(∆M+M\right)}$$

(4) $${\left|\frac{X_{1}}{X_{2}}\right|}_{\omega_{1}}=-\frac{1}{2}\left[\frac{∆M\left(K+K_{c}\right)+\sqrt{∆M^{2}{\left(K+K_{c}\right)}^{2}+4MK_{c}^{2}\left(∆M+M\right)}}{K_{c}\left(∆M+M\right)}\;\right]$$

(5) $$\omega_{2}^{2}=\frac{\left(K+K_{c}\right)\left(∆M+2M\right)-\sqrt{∆M^{2}{\left(K+K_{c}\right)}^{2}+4MK_{c}^{2}\left(∆M+M\right)}}{2M\left(∆M+M\right)}$$

(6) $${\left|\frac{X_{1}}{X_{2}}\right|}_{\omega_{2}}=-\frac{1}{2}\left[\frac{∆M\left(K+K_{c}\right)-\sqrt{∆M^{2}{\left(K+K_{c}\right)}^{2}+4MK_{c}^{2}\left(∆M+M\right)}}{K_{c}\left(∆M+M\right)}\;\right]$$

where ω_1_ in Equation (3) represents the resonant frequency of the individual resonators when the coupled resonators follow out-of-phase vibration and ω_2_ in Equation (5) denotes the resonant frequency of the individual resonators when the coupled resonators follow the in-phase vibration. Furthermore, to obtain the expressions (3)–(6), the masses of the two resonators are assumed to be equal (M_1_ = M_2_ = M) and the stiffness of the two resonators are also assumed to be equal (K_1_ = K_2_ = K) under no perturbation or symmetrical or initial conditions. ΔM represents the mass perturbation that is introduced to break the symmetry of the system and Kc represents the coupling stiffness between the resonators. Equations (4) and (6) represent the out-of-phase and in-phase amplitude ratio obtained at the resonant frequencies ω_1_ and ω_2_ for the coupled MEMS resonators by considering the ratio of the amplitudes of individual resonators, respectively. When considered as an array, the mass and stiffness values for the resonator array can be calculated based on individual resonators in the system as shown in [45],

(7) $$M_{array}=M_{1}+M_{2}$$

(8) $$\;\;\;K_{array}=K_{1}+K_{2}\;\;\;$$

(9) $$\;\;\;C_{array}=C_{1}+C_{2}\;\;\;$$

The *Q* of the coupled MEMS resonator array is then obtained by the expression, as given in [45]

(10) $$Q_{array}=n.{\left(\frac{1}{Q_{1}}+\frac{1}{Q_{2}}\right)}^{-1}$$

where *n* is the number of resonators in the array.

## 4. Results

### 4.1. Sensitivity Analysis

The open-loop frequency response measurements of the coupled MEMS resonators recorded using a two-channel Lock-in amplifier (HF2LI) over the soot particle impaction is used for analysing the sensitivity of the coupled MEMS resonators to soot particles. The frequency response measurements are obtained before and after soot particle impaction at different time intervals for about 40 min. Since the vibrational response of the coupled MEMS resonators at the out-of-phase mode will be dominant when the system is driven via only one resonator as shown in Figure 2, the out-of-phase coupled Lamé mode with the resonant peak centred around 2.508 MHz is considered as the desired mode of transduction in this study.

**Figure 7:**
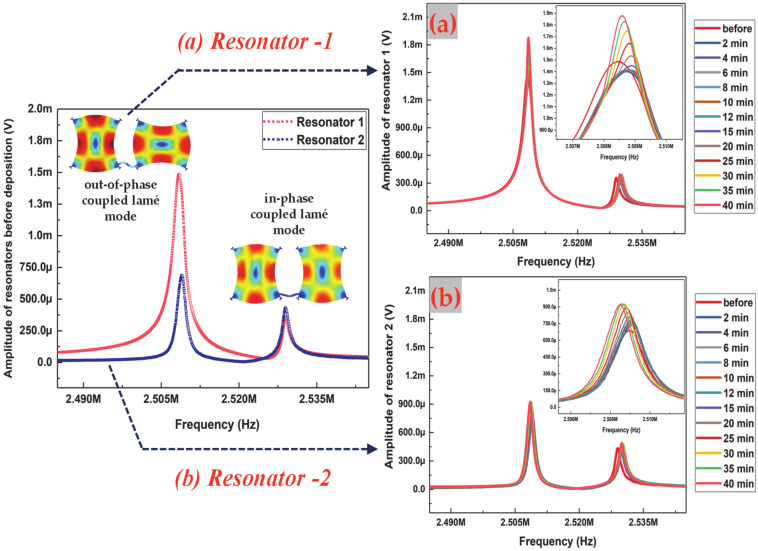
Frequency response of the coupled MEMS resonators monitored using lock-in amplifier before soot particle impaction; (**a**) Frequency response of resonator 1 and (**b**) Frequency response of resonator 2, monitored using lock-in amplifier for 40 min of soot particle impaction at different time intervals on one of the coupled resonators.

Figure 7 shows the open-loop frequency response of the device where the response peak centred around 2.508 MHz corresponds to the out-of-phase mode of vibration in the coupled MEMS resonators. This open-loop frequency response is obtained by performing a frequency sweep around 2.508 MHz using a frequency span of 60 kHz with 6001 data points. The resonant peak centred around 2.53 MHz corresponds to the in-phase mode of vibration in the coupled MEMS resonators. The amplitude ratio obtained by measuring the motional currents between the individual resonators at the out-of-phase mode of vibration in the coupled MEMS resonators, before soot particle impaction is around 2.159 as shown in Figure 7. As shown in Figure 7a,b, the open-loop frequency response of the coupled MEMS resonator is obtained by monitoring the resonant peaks centred at the out-of-phase and in-phase coupled Lamé modes of resonator 1 and resonator 2, simultaneously over the 40 min of soot particle impaction on one of the coupled resonators at different time intervals. The inset in Figure 7a shows the amplitude variation of resonator 1 at the out-of-phase coupled Lamé mode. Similarly, the inset in Figure 7b shows the amplitude variation of resonator 2 at the out-of-phase coupled Lamé mode. It should be noted that the time interval of soot particle impaction over the total 40 min is not uniform throughout the experiment.

In practice, for each time interval of soot particle impaction, the vacuum pump at the exhaust is switched on and off to regulate the deposition of particles onto one of the coupled MEMS resonators. With the initial 2-min time interval of soot particle impaction, it is observed that the time is not sufficient for the stabilization of the soot particle generator. Meantime, in the 2-min time interval of soot particle impaction, a sudden change in temperature, humidity, and pressure is created within the impactor volume because of the airflow regulated by the nozzle to draw particles towards the resonator. These combined effects also influence the resonator and the resonator response is dominated by these effects instead of the mass loading due to the deposited particles.

In addition, both the effective mass and effective stiffness of the resonator will be affected when particles deposit onto one of the coupled MEMS resonators. This could occur under two conditions depending on the stability of the soot particle generator. Firstly, if the deposited particles are densely packed as clusters, the Young’s modulus of the particle clusters is likely to be different from that of the resonator. This could create a dominant stiffness change effect in the resonator rather than effects due to mass change. Secondly, if the deposited particles are sparsely packed as individual spherical particles, then the attachment onto the resonator surface might be loose. Depending on the attachment of particles onto the resonator surface as shown in Figure 5c,d, the frequency response of the resonator could vary. The effects due to particle attachment are likely to dominate in the initial period of 2-min impaction when the generated soot particle concentration is maintained low (~10^3^ particles/cm^3^) as in the case of this experiment.

Initially, a number of deposition experiments are conducted to assess the specific time period over which measurements stabilise sufficiently so that the response is dominated by mass loading. In these experiments, soot particle deposition was conducted at further 2-min time intervals up to 12 min. Eventually, the time interval of soot particle impaction is increased to about 3-min and then 5-min. At the 20th minute of soot particle impaction, and with an increase in the time interval of soot particle impaction to about 5 min, it is observed that the soot particle generator began to stabilise. The measurements then obtained after the 20th minute of soot particle impaction seemed to be dominated by the mass loading effects as there are no significant fluctuations in frequency, which are observed in the time period until 20 min of soot particle impaction.

**Figure 8:**
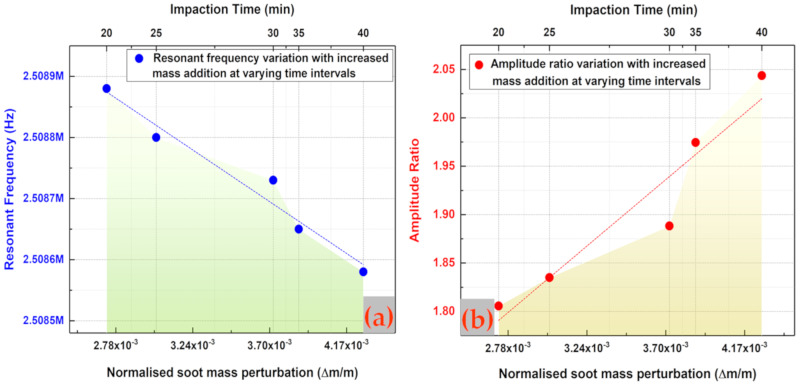
(**a**) Resonant frequency shift and (**b**) Amplitude ratio shift of the coupled MEMS resonators for 20 min of soot particle impaction at different time intervals on one of the coupled resonators following the initial 20 min stabilization period.

**Figure 9:**
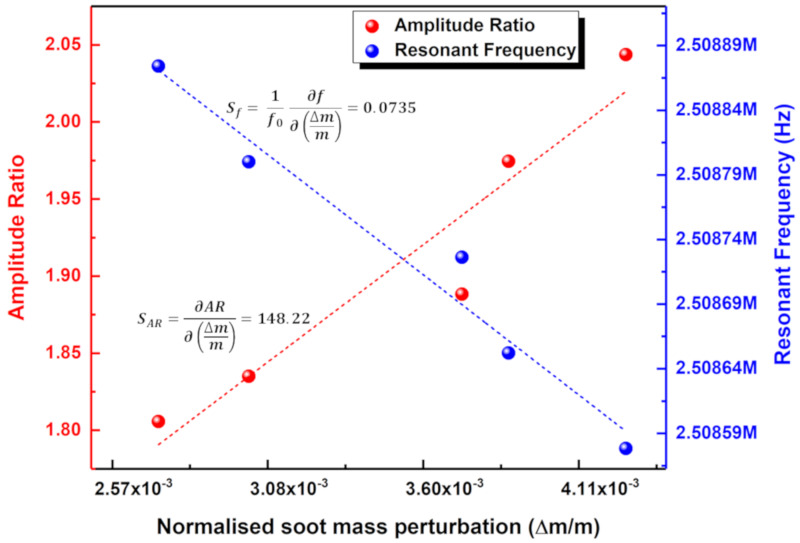
Sensitivity of the coupled MEMS resonators based on amplitude ratio shift and resonant frequency shift for the second 20 min of soot particle impaction on one of the coupled resonators.

The measurement obtained at the 20th minute of soot particle impaction is, hence, considered to be the initial condition/baseline for further frequency response measurements. The resonant frequency shift for one of the coupled resonators (resonator 1) and the amplitude ratio shift in the coupled resonators are then plotted as shown in Figure 8, for a 5-min time interval of impaction until 40 min. It can be observed from Figure 8a that the resonant frequency shift of one of the coupled resonators (resonator 1) decreases during the period of aerosol impaction following the baseline measurement at the 20th minute of impaction. Similar to the trend in resonant frequency shift, it can be observed from Figure 8b that the amplitude ratio shift of the coupled resonators increases for the 20 min of aerosol impaction following the baseline measurement at the 20th minute of impaction. To compare the sensitivity of the coupled MEMS resonator based on amplitude ratio shift and resonant frequency shift for the 20 to 40 min of soot particle impaction on one of the coupled resonators, Figure 9 is then plotted with added soot particle mass as the input variable. 

It can be seen from Figure 9 that the sensitivity to added fractional mass based on amplitude ratio shift is three orders of magnitude higher than the corresponding sensitivity based on resonant frequency shift. It should also be noted that the amplitude ratio has increased from 1.8 to 2.05 for about 20 min of impaction, which in turn indicates that the amplitude ratio of the coupled resonators is highly sensitive to the soot particle mass impacted onto the resonators, even at the maintained lower concentration. This confirms the principle of vibration mode localisation employed in the weakly coupled MEMS resonators to enhance the sensitivity of the MEMS sensors to the added soot particle mass.

### 4.2. Quality Factor of the Coupled MEMS Resonator Array

**Figure 10:**
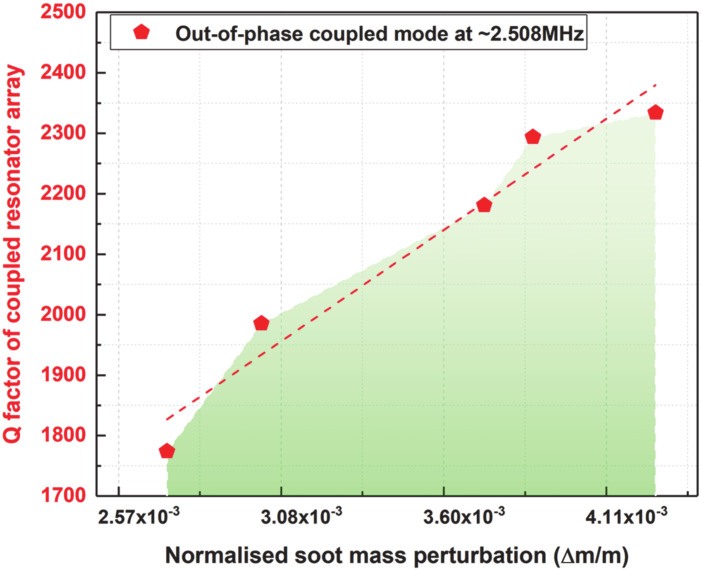
Quality factor of the coupled MEMS resonator array for the second 20 min of soot particle impaction at different time intervals on one of the coupled resonators.

The Q factor of the coupled MEMS resonator array is plotted based on Equation (10) by considering the 20th minute of soot particle impaction as the baseline measurement, as explained in the previous section. The Q factor is obtained for both the resonators (resonator 1 and resonator 2) based on 3dB bandwidth from the open-loop frequency response measurements recorded in Figure 7a,b. The Q factor of the coupled MEMS resonator array with increased soot particle loading from the 20th minute of soot particle impaction until the 40th minute is then plotted, for the coupled resonators operating at their out-of-phase coupled Lamé mode in the study. Figure 10 shows the Q factor of the coupled MEMS resonator array with increased soot particle accumulation on one of the coupled resonators. The Q factor of the array at the 20th minute of soot particle deposition is found to be 1773.8 and the Q gradually increases to 2350, as can be seen from Figure 10. It can be observed that the Q factor of the coupled MEMS resonator array is high enough to enable high resolution and stable mass measurements with increased soot particle loading. 

### 4.3. Stability Analysis

The closed-loop measurements of the coupled MEMS resonators recorded using a two-channel lock-in amplifier (HF2LI) enable estimation of the device stability. Closed-loop measurements are obtained for both amplitude ratio and frequency without soot particle impaction for about 12 h at the out-of-phase coupled Lamé mode in the study. Though the vibrational response at the out-of-phase coupled mode is considered for stability analysis, it can be assumed that in a symmetrical system, the in-phase coupled-mode stability will be similar to the out-of-phase coupled-mode stability [32].

**Figure 11:**
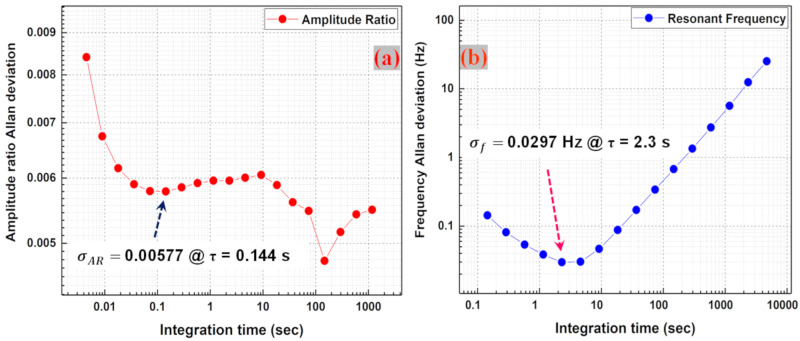
(**a**) Measured amplitude ratio Allan deviation of the coupled MEMS resonators. Best amplitude ratio stability of 0.00577 occurs at τ = 0.144 s, (**b**) Measured frequency Allan deviation of the coupled MEMS resonators. Best frequency stability of 0.0297 Hz occurs at τ = 2.3 s.

The Allan deviation of the amplitude ratio and the resonant frequency for the weakly coupled MEMS resonators is then calculated from the amplitude ratio and frequency data recorded in closed-loop established using PLL function in HF2LI. The long-term stability of the coupled MEMS resonator system is thus investigated based on a comparison between the stability of the two-output metrics. Figure 11a shows the absolute stability of the amplitude ratio measurements in the coupled MEMS resonators. The trend of the amplitude ratio curve shows that the amplitude ratio is more stable at higher integration times with a plateau reached in the period between 0.1–1000 s or so with the representative amplitude ratio stability of ($\sigma_{AR}\;$) 0.00577 achieved for an integration time of 0.144 s. Figure 11b shows the absolute stability of resonant frequency and the trend of the curve shows that the frequency stability decreases with larger integration times with the best stability of ($\sigma_{f}\;$) 0.0297 Hz obtained at an integration time of 2.3 s, due to drift in the resonant frequencies. This makes it evident that the amplitude ratio output metric is more suitable for long-term PM measurements compared to the resonant frequency output metric.

The resolution of the MEMS particulate mass sensor (weakly coupled MEMS resonators) is governed by the input referred stability though the amplitude ratio stability and frequency stability are important output metrics [32]. The input referred stability in terms of normalized mass perturbation in the coupled resonator system, created by soot particle impaction, $\sigma_{\delta M}$, signifies the minimum mass perturbation that can be sensed by the weakly coupled MEMS resonator system. The input referred amplitude ratio stability and frequency stability can be calculated as:

(11) $$\sigma_{\delta M,AR}=\frac{\sigma_{AR}}{\left(\frac{∆AR}{∆m}\right)}$$

(12) $$\sigma_{\delta M,f}=\frac{\sigma_{f}}{\left(\frac{∆f}{∆m}\right)}$$

**Figure 12:**
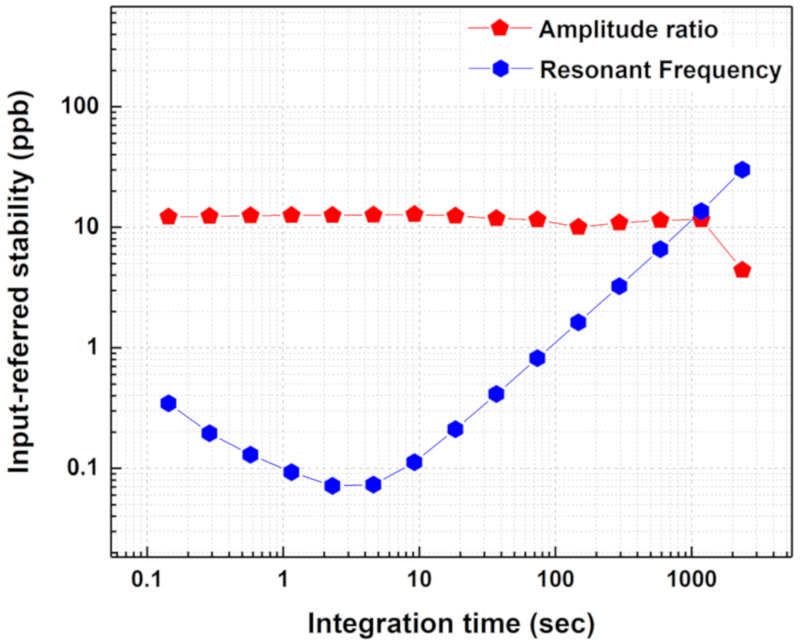
Estimated input referred stability utilising the sensitivity values indicate that the amplitude ratio output metric is suitable for long-term measurements.

Equations (11) and (12) determine that the best input referred amplitude ratio stability of $3.678\times 10^{-10}grams\;$occurs at τ = 0.144 s and the best input referred frequency stability of $2.1619\times 10^{-12}grams$ occurs at τ = 2.3 s. In other words, the minimum mass that could be resolved based on the amplitude ratio shift output readout is calculated as 367.8 picograms and that which could be resolved based on the resonant frequency shift output readout is calculated as 2.16 picograms. This shows that the minimum mass perturbations created in the coupled MEMS resonator system by soot particle impaction can be resolved by both frequency and amplitude ratio for shorter integration times. However, for long-term measurements (in this case τ > 1000 s), the amplitude ratio shift output metric provides more stable measurements and a better resolution than the resonant frequency shift output metric as shown in Figure 12.

## 5. Discussion

### 5.1. Comparison of Amplitude Ratio Shift and Resonant Frequency Shift Output Metrics

The sensitivity and stability values based on amplitude ratio and the resonant frequency of the coupled MEMS resonator system has been utilised to estimate the minimum mass that could be sensed using the vibration mode localized weakly coupled MEMS resonator system. This shows that at shorter integration times (τ < 1000 s), both amplitude ratio shift and resonant frequency shift could resolve the minimum detectable mass. However, for longer integration times (τ > 1000 s) the amplitude ratio output metric is more stable than the resonant frequency shift output metric. This proves that the amplitude ratio output readout is suitable for long-term measurements and also enables better resolution within the same device in this study. Most of the MEMS particulate measurement systems that have been employed so far to detect the particulates (smoke, cigarette particle, etc.,) utilise resonant frequency shifts as the output metric. It is noted that the amplitude ratio shift output metric in the weakly coupled MEMS resonator system together with its intrinsic common-mode rejection as reported in [31] could be an attractive candidate for use in chip-scale particulate MEMS sensors compared to the resonant frequency shift output metric-based sensors.

### 5.2. Comparison of MEMS-Based Mass Estimation With the CPC Inferred Mass

**Figure 13:**
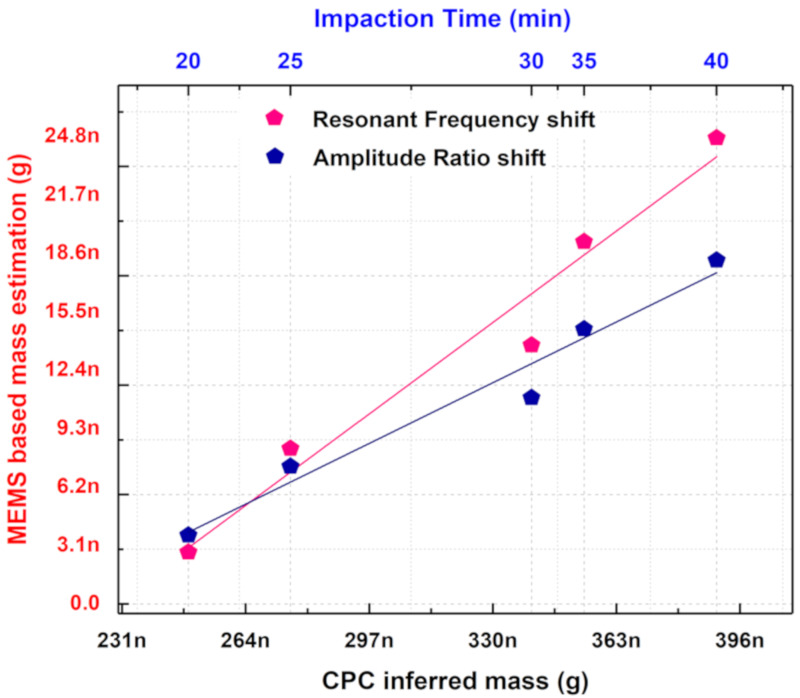
Soot particle mass estimated using coupled MEMS resonators and condensation particle counter (CPC) reference for 20 min of aerosol impaction.

To compare the efficiency of the coupled MEMS resonators in sensing aerosols with that of the reference instrument, the soot particle mass indirectly inferred by the CPC versus the soot particle mass estimated by the MEMS sensor is plotted in Figure 13. A clear correlation is seen between the CPC inferred mass and the mass estimated based on the MEMS sensor indicating the potential for the MEMS sensor to be calibrated with respect to an established reference instrument. The CPC reference instrument displays the particle number concentration of the generated soot particles as the output. To obtain CPC inferred mass, the particle number concentration monitored by the CPC is converted to particle mass concentration based on the equation given in [46]. 

Using the flow rate and time of impaction, the calculated mass concentration values are converted into soot particle mass. This is then compared with the mass estimated by the MEMS sensor based on amplitude ratio shift and frequency shift. A loss factor of 7.143 could be seen between the mass estimated based on frequency shift and the CPC inferred mass from Figure 13. A similar loss factor of 10.246 could be seen between the mass estimated based on the amplitude ratio shift when compared to the CPC inferred mass. The deviation in the mass estimated based on amplitude ratio shift and the mass estimated based on frequency shift could arise from the spatial sensitivity effects of the MEMS resonator. The spatial sensitivity of the MEMS resonator could significantly influence the amplitude ratio output metric. Note that the non-uniformity in soot particle deposition as shown in Figure 5c,d could also contribute to the deviation in the mass estimated based on amplitude ratio and frequency. Furthermore, Figure 13 also shows that the generated soot particles have not been completely impacted onto the MEMS resonators and instead lost by different mechanisms within the MEMS Impactor Stage.

### 5.3. Limitations of the MEMS Sensor

The weakly coupled MEMS resonators employed for aerosol sensing in this work can demonstrate enhanced parametric sensitivity and long-term stability based on amplitude ratio readout. Besides, these resonators can also demonstrate the potential for common-mode rejection to temperature as reported in [31]. However, common-mode rejection is not possible when the resonators are subjected to airflow as only one of the resonators can be treated to airflow at a time. Correspondingly, the mass resolution based on amplitude ratio measurement is lower compared to mass resolution based on frequency measurement. This can be further improved by improving the amplitude resolution of the measurements by optimizing the measurement interface electronics including designing suitable low-noise front-end preamplifiers. Most often, the initial asymmetry created in the frequency response of the coupled MEMS resonators due to fabrication tolerances is treated as a disadvantage as it requires tuning of frequency. Nonetheless, the MEMS sensors based on the mode localisation approach can achieve mass balancing condition when a periodic deposition is carried out in both the resonators as described in [31]. This, in turn, overrides the need for initial frequency tuning as mass balancing could potentially aid in extending the lifetime of the sensors. Although an efficient solution for periodic cleaning is crucial for the practical realisation of a competing integrated chip-scale MEMS particulate sensor, the mass balancing mechanism together with other cleaning approaches adds to the advantage rather than a limiting factor. Furthermore, the spatial sensitivity of the desired bulk acoustic mode and spurious mode coupling effects reported in these bulk acoustic piezoelectric MEMS resonators [47,48], could challenge accurate absolute mass estimation. Nonetheless, these effects can be overcome by operating at resonant modes offering near-uniform spatial sensitivity or by employing annexed sensing platforms [49], shaping the transducer electrodes efficiently for the enhancement of the desired mode of vibration and suppression of the spurious modes [50].

### 5.4. Improving the MEMS Sensor Parameters

To improve sensitivity and resolution, the coupled MEMS resonator dimensions could be scaled down, which would then yield higher frequencies, as they are governed by side length of these resonators [51]. Excessive scaling could further lead to adsorption-desorption noise, temperature fluctuation noise, and insufficient power handling. Even though limitations related to noise issues could be mitigated under the right pressure and temperature conditions, power handling would have to deal with impedance matching problems. However, by making use of coupled MEMS resonator arrays, it would be possible to combine multiple high impedance resonators to allow impedance matching to a much lower value [52,53]. Note that when the vibration mode localisation principle is used for enabling the output transduction approach, a further increase in sensitivity can be obtained when the number of coupled resonators is increased [54].

On the other hand, the reduced surface area offered by scaling MEMS resonator dimensions would give rise to a smaller surface area for interaction of particles with the resonators. This would pose a limitation for long term PM measurements once when the active surface area of the resonator is filled with particles. Despite that, the ability of weakly coupled MEMS resonators to achieve mass balancing, as reported in [31], could increase the sensor lifetimes and thus enable long-term PM measurements.

## 6. Conclusions

This paper provides an experimental demonstration of the detection of ultra-fine particulate matter by vibration mode-localized MEMS gravimetric sensors. A device based on weakly coupled piezoelectric MEMS resonators is shown to sense diesel soot particles of approximately 100 nanometre diameter. The results are in alignment with measurements made using a condensation particle counter. Both resonant frequencies and resonator amplitudes are simultaneously tracked and both measurements are compared. It is seen that the amplitude ratio shift output metric in the coupled MEMS resonators provides high sensitivity to adsorbed particulates. Together with its innate common-mode rejection capabilities as reported in [31], the coupled MEMS resonators employing amplitude ratio readout is an attractive candidate for long-term real-time particulate measurements.

In future work, particle sizing can be conducted by designing a separation system using custom air-microfluidic channels on these coupled MEMS resonators such that each resonator, in principle, could serve as a particle collection bin based on varied particle sizes. The design could also be improved further to extend the number of resonators in the array such that identification of the different types of particles could be enabled as reported in [55]. Additionally, on-chip implementation can be integrated with signal conditioning & data processing circuits and microfluidics, resulting in microsystems with improved performance and form factor. Along these lines, future extension of the current work will address the compaction of the associated electronics for actuation and readout, as well as the integration of the dual MEMS resonator sensing platform with air-microfluidics to construct inexpensive arrays of resonant MEMS sensors to enable highly multiplexed and real-time particle detection.

## Figures and Tables

**Table 1:** Dimensions of the weakly coupled MEMS resonators.

Parameter	Value
Side length of the square resonator	*L = 1400 µm*
Thickness of the square resonator	*h = 10 µm*
Mass of the resonator array	*m_array_ = 91.336 µg*
Stiffness of the resonator array	*k_array_ = 14.61 MN/m*
Thickness of the AlN film	*h_AlN_ = 500 nm*
Thickness of the Al film	*h_Al_ = 1 μm*
Unloaded Q of the resonator array	*Q_array_ = 1773.8 (out-of-phase)*
